# Exploring obscenity in Nigerian Afro-pop music: A sociocultural analysis of Yoruba youth and social norms

**DOI:** 10.1371/journal.pone.0346533

**Published:** 2026-05-05

**Authors:** Oluwatobi Emmanuel Egbowon, Dilan Çiftçi, Ibrahim Adeshola

**Affiliations:** 1 Faculty of Communication and Media Studies, Cyprus International University, North Cyprus, Turkey; 2 Department of Computer Engineering, Faculty of Engineering, Final International University, North Cyprus, Turkey; 3 UNEC Research Center of Digital Economics, Azerbaijan State University of Economics, Baku, Azerbaijan; University of Pecs Faculty of Humanities: Pecsi Tudomanyegyetem Bolcseszettudomanyi Kar, HUNGARY

## Abstract

Nigerian Afro-pop music is a popular genre that significantly influences Nigerian society and beyond. While Afro-pop promotes Nigerian culture globally, critics argue that its explicit lyrics and provocative music videos negatively impact young individuals, promoting materialism, sexual promiscuity, and drug use. This research focuses on the cultural implications of “vulgar” Afro-pop music among Yoruba youth in Nigeria. The study employs a quantitative analysis of 295 responses to explore the respondents’ perspectives on offensive content in Nigerian Afro-pop music. The findings highlight music’s potential positive and destructive influences on youth and emphasize the need for regulatory bodies to scrutinize music content before distribution.

## 1 Introduction

The ability of music to convey meaning, arouse emotions and influence individuals has been a long-standing phenomenon [1]. It is an incontrovertible truth that, aside from Nollywood, one of the primary cultural commodities emanating from Nigeria presently is music, specifically the genre known as Hip Hop [2]. In Nigeria’s musical context, Afro-pop has emerged as a prominent genre, especially among the youth [3]. However, there is an area that requires examination, such as the impact of Afro-pop Nigerian music lyrics on individuals’ values, specifically in relation to negative influence, vulgarity and morality. The Yoruba ethnic group in Nigeria is renowned for its rich cultural heritage, which encompasses various traditions, values, and moral beliefs, Yoruba culture encompasses a set of stringent rules, norms, and mores that dictate the expected behavioural patterns of individuals [4] within this sociocultural context, the lyrical content of Afro-pop Nigerian music may impact the existing cultural framework and potentially shape individuals’ values and behaviour. Hence, understanding the dynamics of music’s impact on cultural values within this community necessitates understanding Afro-pop music lyrics’ influence on negative influence and morality among Yoruba indigenes.

As a genre in musicology, Afro-pop is a category of hybridised popular music, a fusion of African musical traditions of the continent, especially Afrobeat, Fuji, and Juju, and western music, such as hip-hop, R&B, and electronic music. Afro-pop is musicologically defined (as a genre) as polyrhythmic, based on traditional African drumming patterns, call-response vocal patterns, syncopated percussion, and a focus on dances. The genre uses both live instrumentation (guitars, keyboards, traditional drums) and digital production, and the lyrics are done in a mix of English, Nigerian Pidgin, and native languages, like Yoruba and Igbo [2,5].

Although hip-hop is confused with afro-pop in the Nigerian scenes, the music is unique in terms of musicality. Hip-hop, which originated in the 1970s in the South Bronx, New York, is defined by four main parts, including rapping (rhythmic spoken word on top of beats), DJ’ing (turntablism and beat-making), breakdancing, and graffiti art. The aural trademark of hip-hop is in sampled breakbeats, boom-bap drum patterns, and is based on the lyrical content that is not presented in sung melody but in rap instead [6]. However, in Nigeria, the two genres have gotten deeply intertwined that modern artists have worn the melodic and African rhythmic roots of Afro-pop with the lyrical delivery style and thematic focus of hip hop.

Our analysis will be on the Nigerian Afro-pop genre with reference to artists like Wizkid, Flavor, and Olamide. The representative songs which both reflect the musical features of the genre and the content of the lyrics studied are songs by Olamide (Don’t stop) (banned by the National Broadcasting Commission in 2016 due to obscene content), Flavour (Shake), and the songs with explicit sexual references, materialistic subject and profanity that are the main focus of the given research paper [6,7].

Previous research has explored the impact of music on individuals’ behaviour, emotions, and cultural values, with a focus on its potential to shape societal norms and attitudes. Samson [8] conducted a study on Sexism and power play in the Nigerian Contemporary Hip-hop culture and discovered that song lyrics, especially those containing explicit sexual content, may exploit sexuality and crude characters, influencing the perception and behaviour of listeners. Similarly, [9] emphasized that musical preferences can reflect aspects of listeners’ personalities and have a negative influence on their attitudes. Furthermore, [10] content analysis of popular songs revealed a “contagion effect,” where lyrics reflecting mass psychology trends influenced individuals who were not previously predisposed to such attitudes. While these studies provide valuable insights into the influence of music on individuals, there is a need for research specifically focused on the cultural implications of vulgar Afro-pop Nigerian music among Yoruba Youths, exploring how it shapes their values within socio-cultural contexts that contradict the Yoruba value system.

The purpose of this research is to investigate the negative influence and morality of Afro-pop Nigerian music lyrics among Yoruba Youths. By examining the specific cultural context of the Yoruba ethnic group and their interactions with Afro-pop Nigerian music, this study aims to deepen our understanding of how music shapes individuals’ values and behaviour within socio-cultural contexts that may contradict the prevailing cultural norms. The need to keep traditional moral systems of the Yoruba culture is not simply an issue of cultural choice. Recent world experiences have shown that rapid moral relativism may have certain social outcomes [11]. For example, strong moral relativism has not only created a “slippery slope” in moral standards, but may also have contributed to political and economic instability, including reactionary movements and a weakening of social unity [12]. In the Yoruba context, the loss of traditional values is posing a threat to the social structure, which in the past has been providing the community with stability, continuity between generations, and ethics in the development of the youths. This research aims to give more understanding to the intricate relationship between music and culture and provide valuable insights for both scholars and practitioners in the fields of music psychology, cultural studies, sociology and many others. Moreover, this study will help identify possible implications for the Yoruba community in terms of cultural preservation, youth development, and promoting positive social norms.

## 2 Literature

### 2.1 Yoruba social construct, beliefs and music

The Yoruba people, recognized as one of Nigeria’s largest ethnic groups, possess a noteworthy social framework that is deeply embedded in their customs and traditions. Yoruba society places significant importance on family, with extended familial bonds and kinship connections establishing the foundation of their social composition [13]. Communal values and reverence for elders are highly esteemed, while the notion of “Omoluabi” accentuates the value of good character, ethical principles, and moral righteousness [14]. Nevertheless, Yoruba society grapples with obstacles that stem from urbanisation, modernisation, and socio-economic imbalances [15]. These challenges can potentially strain traditional social configurations and impede community cohesion and advancement. The culture of Yoruba is intricately intertwined with a multifaceted and elaborate spiritual belief system and an assemblage of deities referred to as “Orisha.” The Yoruba people harbour a resolute conviction in the supernatural and the presence of various gods, each of whom is associated with specific facets of life [16]. These beliefs serve as the bedrock for cultural identity and practices, albeit they can engender tension within a rapidly transforming society. Globalisation, religious pluralism, and the impact of modern ideologies pose challenges to conventional Yoruba beliefs and practices [17]. These dynamics are capable of instigating debates and conflicts regarding the preservation of cultural heritage and the adaptation of Yoruba beliefs to modern contexts. Music in Yoruba culture serves as a means of communication, social dialogue, storytelling, and cultural expression [18]. Traditional Yoruba music is characterized by rhythmic complexity, vibrant drumming, and intricate vocal harmonies. It plays a vital role in religious ceremonies, social gatherings, and cultural festivals, contributing to a sense of community and identity [19]. However, challenges to the preservation and promotion of traditional Yoruba music arise from the modern music industry and changing tastes. the commercialisation of music, Western influences, and the dominance of popular music genres can diminish the cultural significance of traditional Yoruba music, thereby jeopardizing its transmission to future generations [5]. Efforts are being made to address these challenges through initiatives that promote and preserve Yoruba musical heritage.

### 2.2 Obscenity and vulgarity in contemporary Nigerian Afro-pop music

To analyze the exact influence of vulgar Afro-pop on Yoruba youth, it is imperative to set some deeper context for the nature and direction of the obscenity in the Nigerian popular music scene. Obscenity in this sense includes various behavioural tendencies and expressions such as sexually explicit lyrics, cussing, degradation of women, and promotion of deviant behaviour, which transgress the moral and cultural boundaries of the Nigerian society [6,20]. Knowledge of the development of such content, how it is represented in particular artists and songs, and why regulatory tools have been unable to control it, gives us a proper base to examine this issue as it applies to the Yoruba youth in later sections.

Nigerian popular music, generally categorised as Afro-pop music, is a hybridised genre; a mixture of native African musical styles, more specifically, Afrobeat and Fuji, Juju and Highlife, under the influence of western music, mainly hip-hop, R&B and electronic music [2]. Afro-pop and hip-hop have been used interchangeably in the Nigerian setting; however, they are musically different. As a movement that started in New York in the 1970s, hip-hop is characterized by four basic forms: rapping (MCing), DJing, break dancing, and graffiti art, which was traditionally used as a tool of social commentary and political action by marginalized groups [6]. The Afro-pop, in turn, is a more expansive, more commercial genre with its foundation in the African sonic identity, and it is marked by the polyrhythmic patterns, the call-and-response vocal patterns, and the focus on the African cultural aesthetics [2]. The two genres are now inextricably mixed with modern artists, including Wizkid, Davido, and Olamide, taking the Afro-pops soul and the hip-hop poetry and lyrics with subject matter in their music. Not the most disturbing cases of obscenity and vulgarity have been raised without this mixed genre.

The theme of the early Nigerian hip hop of the late 1980s and 1990s was relatively socially aware, dealing with youth unemployment, governmental corruption, and social inequality [21]. Towards the end of the 1990s and especially in the 2000s, the genre underwent a substantial change in theme. Under the influence of what was defined by De Castro (2007, as cited in Oniwon and Salami [6]), a mass culture of consumerism, Nigerian popular music began to embrace materialism, sexual promiscuity, and obscenities as necessary techniques of aesthetics and commerce. In a content analysis of 178 hip hop music videos in Nigeria, published between 2011 and 2016, Oniwon and Salami [6] reported that the materialistic, vulgar, and profane themes were extremely prevalent in the sampled information. They found that in 43.26% of the videos sampled, they found materialistic lyrics between 16 and 20 instances per song, and profane language was found 21 times or more in 42.7% of the videos sampled. The implication of this quantitative result is impressive. Obscenity in Nigerian popular music is not an isolated case but a mere part and parcel of the commercial formula of the genre.

The particular shapes through which the vulgarity is expressed in the Nigerian Afro-pop is complex. Oniwon and Salami [6] recognize three key types, namely materialism, which deals with the glorification of wealth, luxury goods, and conspicuous consumption, vulgarism, which deals with sexually offensive or degrading colloquial expressions, and profanity, which is the use of strong, foul, and swearing language. In the case of vulgarism specifically, they analysed the 35.39% of videos sampled that had expressed vulgar lyrics 16–20 times, and 32.02% expressed vulgar content at least 21 times or more. Most importantly, the researchers established that the implicit meaning of this vulgarism always reflected the objectification of female bodies. In Olamide and his popular song Falila Ketan, to mention just one example, the lyrics put in direct context the mode of dress and physical appearance of a woman as a request for sexual penetration. This tendency of male gratification through the use of the female body as the lyrical object is a repeat of the genre.

Any discussion of obscenity in Nigerian popular music cannot be discussed without referring to the analysis of individual artists whose efforts have become the paradigm of this trend, especially in the same Yoruba-speaking culture of music. The example of Abass Akande Obesere and Janet Ajilore, Saint Janet, especially teaches the Yoruba setting. Ademowo and Balogun [22] used Foucault’s theory of sexuality to discuss how these two artists injected explicit sexual discourse into the hitherto conservative Yoruba musical arena of Fuji and Juju music. Obesere, with his own self-declared title of Oba Asakasa, meaning ora, master of lewd songs, had made his entire career on violating the Yoruba cultural taboo on discussing sex in public. His words, as recorded by Ademowo and Balogun [22], are written in an euphemistic but very clear Yoruba language explaining sexual organs and sexual acts in a manner that was never heard in the traditional Yoruba performance. This was also true of Saint Janet, who introduced vulgar sexual content into the Juju genre with parodies of Christian worship songs as the bearers of sexual suggestiveness. What is especially noteworthy about this discovery is that it has shown that the emergence of explicit content in the Yoruba popular music was not solely a product of the influence of Western hip-hop, but it also arose internally in the tradition of Yoruba music, and therefore, a more intricate history of obscenity than commonly described.

The Nigerian Afro-pop sexuality does not involve innuendo and euphemism. Ojoawo and Akande [23] researched the sexual metaphors by employing a hip-hop group in Nigeria, where the artists employ an elaborate use of linguistic tactics, such as a double entendre, slang, borrowed words in Nigerian languages, so that they can pass over sexual messages without total denial. This linguistic complexity implies that the real sexual implications of numerous songs might not be clear to the uninitiated audience who do not have profound knowledge of young Nigerian slang, but to the target audience’s main participants, young Nigerian listeners. Multiplicity of Nigerian languages of Yoruba, Igbo, and pidgin English, used in one song, as it is recorded by Oniwon and Salami [6] in their evaluation of Olamide lyrics, therefore allows performers to incorporate sexually explicit materials in a manner to avoid regulation controls to some extent. Meanwhile, Omobowale et al. [24] considered the work of St. Janet and Olamide (Badoo), who are creating a cultural normality where the value of women is diminished to sexual availability and physical qualities, a trend that can be compounded by the wider trend of gendered objectification that the genre plays.

Regulatory control efforts notwithstanding, the popular music on the Nigerian scene is mostly dominated by obscene content, as it has been widely documented. Endong [20] gives a vivid evaluation of the music censorship systems and structures in Nigeria and says that the obscenity has become so institutionalised in the production of music in Nigeria that it is now being viewed as a tradition and not a deviation. The main regulatory organ, the National Broadcasting Commission (NBC), has the right to prohibit songs on radio and TV airing and exercised this right in such high-profile issues, such as the prohibition of Olamide’s Don’t Stop in 2016 due to obscenity, indecency, and profane expression [6]. But according to Endong [20], these broadcast bans are not a sufficient reaction to how much of an issue it is. The spread of digital music distribution services, social media, streaming services, and sharing on mobile phones implies that music that is not allowed on the airwaves has never been blocked by the regulations that are meant to guard the very young people against whom the regulations are supposed to uphold. Endong [20] thus believes that there should be more radical solutions, like the distribution of sexually explicit musical material should be suppressed, and performance of such musical material should also be prohibited in a setting where children could be found, but he recognizes that globalisation and western cultural imperialism have rendered the definition and imposition of the musical obscenity fundamentally difficult in Nigeria.

Another aspect of the cultural influence of modern Afro-pop that should be discussed is the aspect of its propensity to spread foreign religious models, especially Christianity, which may negatively affect the traditional Yoruba spiritual culture. Although it does not solely deal with the issue of obscenity, it has cultural implications in the wider scope of this study that touches on the erosion of the Yoruba culture. According to an analysis conducted by Ademowo and Balogun [22], the brand of music being promoted by Saint Janet has intentionally obliterated the boundaries between Gospel music and sexually explicit music by having the setting and idiom of Christian worship as the means of lewd performance. This blurring is a broader pattern by which the Christian musical and cultural allusions have substituted customary Yoruba spiritual material in popular music, part of the dislocation of indigenous religious and cultural paradigms in the sound environment available to Yoruba young people.

The themes of obscenity and vulgarity in Nigerian Afro-pop music are not mere superficial and incidental phenomena. They are formally anchored in the commercial logic of the genre, they are linguistically advanced in the form of their performance, they are culturally heterogeneous in their sources, and they are ill-regulated in the current regulatory frameworks. It is in this context that the current research paper considers the particular cultural implications of this content to the Yoruba youths, to which the following sections are addressed.

### 2.3 Afro-pop music lyrics and Yoruba youth cultural behaviour

Pop music is commonly perceived as a genre or practice that is tailored to meet the preferences and tastes of its intended audience. It is characterized by its mass appeal and encompasses a diverse range of styles. Furthermore, it can be easily understood by a significant portion of the general public and its appreciation does not necessarily require an in-depth understanding of musical theory [2]. Music holds immense power in shaping civilisations and social structures globally, influencing individuals’ perspectives, dispositions, and motivations across generations [5]. The interplay of “musical and cultural factors” encompasses the diverse cultural elements embedded in music compositions, while cultural influences continuously evolve alongside musical influences [25]. Music and culture have always shared a strong bond [26]. The widespread availability of recorded music, facilitated by advancements in technology, has made it easily accessible, but its ubiquity in certain societies, like Nigeria, has led to it being taken for granted. Kriger [27] stresses that the widespread availability of recorded music, facilitated by advancements in technology, has made it easily accessible to people around the world. Studies have shown that music has the ability to shape our worldviews and exposure to music with positive lyrics can increase feelings of happiness and well-being [28]. On the other hand, Vulgar music elicits corresponding facial expressions in listeners, while upbeat music has the opposite effect, demonstrating the influence of our mental state on sensory perception [29]. Music triggers chemical reactions in the brain, contributing to the physical responses we experience [30]. Moreover, music’s impact on culture and individual behaviour is noteworthy. Historical records attest to music’s role as a catalyst for change, exemplified by civil rights “freedom songs” like “We Shall Overcome” and “Strange Fruit,” which helped dispel prejudice and foster understanding, During the Civil Rights Movement in the United States, music played a crucial role in inspiring and mobilizing activists, and in raising awareness of the injustices faced by African Americans [31]. In today’s world, the influence of music necessitates heightened cultural awareness. It is crucial to consider the cultures we aim to promote through our music, as music possesses the potential to challenge existing beliefs and shed light on overlooked issues [32]. Several previous studies have explored various aspects of vulgarism and inappropriateness, including issues such as the objectification of women, immoral attire, provocative dancing, and the use of derogatory language and swearing [33–35]. The development of Nigerian music witnessed the emergence of absurd contexts and representations through various themes, which attracted significant criticism from scholars [36]. Popoola [37] emphasized the prevalent themes of love and sex in recent Nigerian hip-hop music. They highlighted the need to restructure Nigerian hip-hop, noting its origins in the late 1980s and 1990s as a refuge for youths seeking solace from corruption, crises, unemployment, and currency depreciation [21]. However, it was further argued that Nigerian hip-hop has become highly profane, with offensive lyrics contradicting [38] that emphasize educational purposes traditionally associated with indigenous music. Based on the theoretical framework established above and the empirical evidence from recent studies on Nigerian Afro-pop’s explicit content, we propose three hypotheses that examine both direct and mediated pathways through which music lyrics influence youth behaviour and societal morality. These hypotheses are grounded in social learning theory and cultivation theory, which suggest that repeated exposure to media content shapes attitudes, beliefs, and behaviours over time.

***H1: Exposure to vulgar Afro-pop music lyrics is positively associated with cultural behaviour that deviates from traditional Yoruba social norms among youth (negative cultural inﬂuence).***

### 2.4 The cultural value of music

Many prominent thinkers in the 18th century, such as Condillac, Rousseau, and Webb, posited that the genesis of music is its ability to convey emotion [39]. Consequently, early humans desired to communicate and connect, they developed empathy through communication, as they learned to understand others’ emotions [39]. It is practical to understand modern society by taking into account music’s pervasive impact, so far music is a method through which people and organisations form and express their identities [40]. In contemporary society, music plays an important part in the process by which society subgroups build their identity and position in the world, serving as a metaphor for the capacity of shared experience to unite individuals and their common beliefs. [41]. The musical genres of classical music and Afro-pop are distinct from one another. Afro-pop music is also frequently linked to the African subculture, because a subcultural group’s basic beliefs may include things like a preference for a particular kind of Afro-pop music. The identity of subcultural groups is strongly influenced by music. Petrušić [42] Although the rise of new media has increased Afro-pop music’s influence and reach in the growth of popular culture and the arts, more must be done to raise the intrinsic worth of music listening. mass culture enjoyed gradually growth as a result of the rise of mass media and the entertainment sector [43]. This results in music losing its philosophical underpinnings and significance and becoming one of a long list of commodity products created by the entertainment business, which aims to homogenize and obliterate the differences between musical genres [44]. The claim that there is an excessive amount of sexual content in Afro-pop song lyrics is not new. Additionally, the idea that people should be cautious about what they hear, especially if they are young people, is not a novel one. Concerns about adolescent listening habits, such as what they choose to listen to, the messages they get, and their vulnerability to influence, have increased recently. Given how crucial Afro-pop music is to the development of adolescents’ identities, it makes sense to be concerned about its sexual content. According to [45], the vital element of the persuasive impact of musically delivered storytelling is the ability to create a “virtual experience” for listeners that amplifies artists’ opinions. This could be a problem when it comes to explicit sexual lyrics that youth listeners might internalize and act upon since they could introduce, promote, or reinforce negative attitudes and actions. Afro-pop music is one of the most striking facets of this generation’s culture. Afro-pop music has represented the cultural changes of young people ever since commercial music emerged. Most Afro-pop songs’ lyrics and music videos discuss a variety of negative issues, including sexual promiscuity, drug usage, gun violence, inebriation, vandalism, drug dealing, money, defiance of authority, fraud, and many more [46]. This in turn influenced the cultural behaviour of the youths. As a result, we hypothesized that:

***H2: Yoruba youth cultural behaviour inﬂuenced by Afro-pop music is negatively associated with adherence to traditional Yoruba moral values in society.***

### 2.5 Lyrics as cultural mirrors

Research analysis conducted by [2] on the lyrical content of Afro-pop songs has revealed an enduring presence of profane themes, such as immorality and fraud, among others. This prevalence of vulgarity in Afro-pop music is not confined to a particular period and encompasses various subgenres within the Afro-pop genre. Notably, the connection between musical genres such as hip-hop and the expression of vulgarism may stem from our shared cultural context, as posited by [47]. The gradual decline of upbeat lyrics reached a plateau in the mid-1990s, while societal and economic pressures have resulted in an emphasis on significant themes, comfort, and romantic sentiments in song lyrics [48]. Criticism against contemporary popular music often centres on the potential influence of lyrics on young listeners’ thoughts and actions, raising concerns about violence, fraud, sexual misconduct, racism, bigotry, Satanism, and substance abuse [49]. Afro-pop music is believed by many to promote antisocial behaviours such as drug abuse, risky sexual conduct, and physical violence. While some Afro-pop songs may advocate for peaceful coexistence, Hip-pop openly glorifies aggression as an exciting lifestyle, this trend is a reflection of the artists’ backgrounds and communities, where a culture of oppositional resistance has emerged in response to the pervasive despair that characterizes their communities [50]. Research indicates that Afro-pop fans, particularly youths, exhibit more aggressive tendencies, drug abuse, excessive smoking, alcoholism, skimpy dressing, and vulgarism while watching and listening to popular music through social media and other avenues [3]. Notably, not all Afro-pop contains explicit immoral content that impacts listeners’ sexual behaviour. However, certain Afro-pop music videos feature sexually explicit lyrics, influencing young people to engage in risky conduct and endorsing such behaviour among peers. Sarah and Savannah [51], in their findings stressed that exposure to sexual content in music videos has been linked to more permissive attitudes toward sexual behaviour and increased acceptance of sexual violence, exposure to sexual content in music videos can increase sexual risk-taking behaviour among young people, including earlier sexual debut, increased number of sexual partners, and decreased use of contraception. Afro-pop is also associated with drug abuse, as lyrics endorse financials scams, and valorisation of drug abuse [52]. Exposure to vulgar music has been linked to negative attitudes and behaviours. The song by Afro-Pop artist namely Flavor titled “Shake,” often known as “Ukwu Nwanyi Owerre,” is characterized by lyrics that revolve around trivial matters, explicit sexual references, provocative stage performances, nudity, and derogatory language that is morally reprehensible [7]. Research also suggests a correlation between exposure to vulgar music and endorsement of sexualisation of women, and the tendency of objectifying women [53]. Exposure to crude language in music has been linked to an increase in sexual curiosity and an earlier initiation of sexual activity among young individuals, according to studies conducted by [35] and [54]. These studies indicate that the sexualisation of young individuals through the medium of vulgar music lyrics may have detrimental effects on their sexual health and overall well-being. As a result, we hypothesized that:

***H3: Exposure to vulgar Afro-pop music lyrics is directly associated with erosion of traditional moral values in Yoruba society, independent of individual cultural behaviour changes.***

## 3 Research methodology

### 3.1 Sample, measurement scale, and data collection

Two hundred and ninety-five participants in this study are Yoruba indigenes who have listened to Afro-pop Nigerian music. Participants were speciﬁcally asked about their exposure to popular Afro-pop artists such as Wizkid, Flavour, Olamide, and others whose music typiﬁes the genre’s contemporary form. As shown in S1 Appendix A, the measurement items for data collection consisted of fifteen items based on three constructs Afro-pop Music Lyrics, Negative Influence, Morality on Society/ Sociocultural value. To reduce bias, the measurement items were culled from multiple sources. The measurement items have been tailored for the study and scored on a 5-point Likert scale ranging from (1) strongly disagree to (5) strongly agree. The study also used convenience sampling, and the questionnaire was sent via Google Forms. Furthermore, respondents took part in the study voluntarily, and their anonymity was ensured. Over the course of two months, 295 replies were obtained. Table 1 presents the demographic profile of respondents.

**Table 1:** Demographic information.

Age Range	Female	Male	Female Frequency	Male Frequency	Total
18-22	23%	17%	34	26	60
23-27	24%	31%	35	47	82
28-32	34%	33%	50	50	100
33-35	18%	18%	26	27	53
**Total ->**			**145**	**150**	**295**

The measurement items were developed based on established scales from [55,56], and [57,58] (see S1 Appendix A), and were adapted to specifically address the Yoruba cultural context. The construct “Afro-Pop Lyrics” measures participant attention to and perception of vulgar language in Nigerian Afro-pop. “Negative Influence” assesses perceived impacts on youth behaviour including violence, disrespect, and objectification of women. “Morality in Society” evaluates perceptions of cultural value erosion and the need for regulatory oversight.

### 3.2 Data analysis and results

Smart-PLS version 4.0.9.0 was used to analyse the data. Smart-PLS was used to test the proposed hypothesized model using the partial least squares structural equation modelling (PLS-SEM) technique [59]. Ringle et al. [60] recommended a two-step procedure. The external measurement model was explored first, followed by the internal structural model. An assessment of the common method bias (CMB) was undertaken prior to the measurement model to determine the magnitude of the CMB. According to [61], the presence of collinearity statistics and a variance inflation factor (VIF) greater than 3.3 suggests pathological collinearity and is a sign that common technique bias may impair a model. Table 2 shows the results of the VIF test; all of the VIF were less than 3.3. As a result, CMB was not severe in our investigation. We used 5000 bootstrapping sub-samples in SmartPLS with the option of individual sign modifications to get inference statistics for the 295 coded items [62].

**Table 2:** Factor loadings.

Constructs	Item(s)	Factor Loading	Outer VIF	Cronbach Alpha	rho_A	Composite Reliability (rho_C)	Average Variance Extracted (AVE)
Afro Pop Lyrics	**AF1**	**0.849**	**2.088**	**0.870**	**0.883**	**0.911**	**0.721**
	**AF2**	**0.894**	**2.684**				
	**AF3**	**0.877**	**2.575**				
	**AF4**	**0.771**	**1.720**				
Negative Influence	**NI1**	**0.850**	**2.880**	**0.857**	**0.860**	**0.905**	**0.706**
	**NI2**	**0.897**	**3.289**				
	**NI3**	**0.895**	**2.786**				
	**NI4**	**0.703**	**1.356**				
Morality in Society	**MS1**	**0.786**	**2.464**	**0.831**	**0.836**	**0.880**	**0.595**
	**MS2**	**0.759**	**2.212**				
	**MS3**	**0.819**	**3.145**				
	**MS4**	**0.747**	**1.851**				
	**MS5**	**0.744**	**1.915**				

### 3.3 Measurement model

The validity and scale reliability of measurement models are evaluated in this section. Performing reliability, convergent, and discriminant validity tests on the model was suggested by [63]. Except for two factors, one for Afro-Pop Lyrics (AF5) and the other for Negative Influence (NI10), all reliability and validity results, as shown in Table 2, indicate that all factor loadings provided are higher than 0.7. Then, we examine the composite reliability (CR > 0.70), Cronbach’s alpha (0.95), rho_A, convergent validity (AVE > 0.5), and discriminant validity of the measures associated with each construct [64–66].

Additionally, the value of AVE varied between 0.595 and 0.721. The AVE value is consequently appropriate, and all items’ AVE values were higher than 0.50, as suggested by [66]. Furthermore, as advised by [65], the composite reliability (CR) was higher than the typical cutoff value of 0.7. Cross-loading values, Fornell and Larcker criteria, and examination of the heterotrait-monotrait (HTMT) ratio to confirm discriminant validity are further checks for the measurement model. The cross-loading values from the results are shown in Table 3, and all loading values are larger than the values for the other similar cross-loading constructs. This demonstrates that the cross-loading results satisfied the criteria set out by [64].

**Table 3:** Cross-loading values.

Items	Afro Pop Lyrics	Morality in Society	Negative Influence
*AF1*	**0.849**	0.382	0.547
*AF2*	**0.894**	0.403	0.595
*AF3*	**0.877**	0.356	0.510
*AF4*	**0.771**	0.364	0.383
*MS1*	0.333	**0.786**	0.308
*MS2*	0.306	**0.759**	0.257
*MS3*	0.284	**0.819**	0.301
*MS4*	0.423	**0.747**	0.378
*MS5*	0.328	**0.744**	0.322
*NI1*	0.443	0.342	**0.850**
*NI2*	0.476	0.363	**0.897**
*NI3*	0.569	0.353	**0.895**
*NI4*	0.530	0.324	**0.703**

AF: Afro Pop Lyrics, MS: Morality in Society, NI: Negative Influence

The square root of the AVE for each construct is shown in Table 4, along with the discriminant validity test for each construct larger than 0.50, as indicated by [64], who also suggested that the AVE should be bigger than the correlation between the constructs and other latent constructs.

**Table 4:** Discriminant validity.

Fornell-Larcker Criterion	Afro Pop Lyrics	Morality in Society	Negative Influence
**Afro Pop Lyrics**	**0.849**		
**Morality in Society**	0.443	**0.772**	
**Morality in Society**	0.607	0.413	**0.840**

Additionally, as shown by the results in Table 5, the HTMT ratio analysis factors met the normative HTMT value threshold of less than 0.9, as recommended by [67]. The findings in Tables 3–5 thus imply adequate and precise findings.

**Table 5:** HTMT ratio analysis.

	Heterotrait-monotrait ratio (HTMT)
**Morality in Society – Afro Pop Music Lyrics**	**0.509**
**Negative Influence – Afro Pop Music Lyrics**	**0.690**
**Negative Influence – Morality in Society**	**0.482**

### 3.4 Structural model

To achieve a correct and good model fit, the study first checked for common method bias and evaluated the multicollinearity among the variables. According to [61], the variance inflation factor (VIF) must be less than 5. According to Table 6, the study reveals that the inner VIF values range from 1.000 to 2.582. The results show that there is no multicollinearity among the variables.

**Table 6:** Collinearity statistics (VIF).

Constructs Relationship	Inner IVF
Afro Pop Music Lyrics – Morality in Society	1.583
Afro Pop Music Lyrics – Negative Influence	1.000
Negative Influence – Morality in Society	1.583

Collinearity was not a problem in our investigation, so the next step was to determine the value of R2 for the endogenous constructs. According to [68], it is crucial to determine the degree of R2 since it measures variance and illustrates the impact of exogenous constructs on endogenous constructs in the model. The R2 value ranges from 0 to 1, with a higher value indicating greater explanatory power. The R2 value for morality in society in this study was 0.229, meaning that 22.9% of the variance in morality in society could be accounted for by its direct effects from exogenous variables (Afropop music lyrics, and negative influence) as well as indirect effects from the mediating relationship of negative influence. The total variances that were accounted for by negative influence were 0.368, indicating that 36.8% of negative influence could be explained by Afro-pop song lyrics. According to [69] basic guidelines for acceptable R^2^, the R^2^ values for the respective targeted endogenous variables in this analysis (negative influence, R^2^: 0.368; morality in society, R^2^: 0.229) were above 0.26, so they were deemed to have demonstrated a significant level of variance. According to [70], the analysis of the predictor weightings on their endogenous variables involves determining the degree of f^2^. According to [69] recommendations, the values of 0.02, 0.15, and 0.35 also represent the endogenous variable’s moderate, medium, and high effect sizes. The analysis’s findings showed that Afropop lyrics had a significant impact on negative influence (f^2^ = 0.583), but only a little one on morality in society (f^2^ = 0.076). Regarding the [69] cut-off, negative influence likewise had a very modest impact on societal morality (f^2^ = 0.043).

Table 7 depicts the connections between the notions that support morality in society. According to the study’s findings, Afro-Pop song lyrics positively impact to negative influences H1 (β = 0.607, p < 0.000). Negative Influence also shows a positive impact on Morality in Society H2 (β = 0.229, p < 0.001). The direct relation of Afro Pop Music Lyrics shows a positive impact on Morality in Society H3 (β = 0.304; p < 0.000). The mediating effect of Negative Influence shows a positive impact on Morality in Society H5 (β = 0.139, p < 0.002). Therefore, empirical evidence supports each of the three hypotheses. As a result, the composite PLS-SEM bootstrapping method revealed that all initial coefficients supported the model’s hypotheses.

**Table 7:** Mediation effects.

IV	MV	DV	Effect of IV on MV	Effect of MV on DV	Direct Effect	Indirect Effect	Total Effect	CI
**Afro Pop Music Lyrics**	Negative Influence	Morality in Society	0.607	0.229	0.304	0.139	0.443	0.327 - 0.556
							0.607	0.522 - 0.687
							0.229	0.096 - 0.366

Independent Variable (IV), Mediating Variable (MV), Dependent Variable (DV), Confidence Interval (CI).

The predictive importance of each endogenous component is examined in addition to R^2^ and f^2^, and all Q^2^ values reported in our sample were significantly greater than zero [71,72]. This shows how the route model can predict its latent variables and then indirectly predict endogenous measurement items using the underlying structural relationships. The model’s result for Q^2^ is 0.183, indicating that it has predictive power for the endogenous construct of morality in society [73]. The cross-validated redundancy shows that negative influence has a predictive capacity with Q^2^ = 0.362. The results demonstrate that for all constructions, the expected Q^2^ values for the PLS-SEM were more significant than zero. As a result, the model’s results for communality and redundancy show that it has a strong capacity for prediction.

## 4 Discussion

This study set out to explore the cultural implications of vulgar Nigerian Afro-pop music among Yoruba youth in Nigeria, with a particular focus on the potential positive and negative impacts. The research aimed to understand the perspectives of respondents regarding offensive content in Afro-pop music and its influence on young individuals. The findings not only validate the concerns raised by critics but also shed light on the complex relationship between music content and its effects on Yoruba youth. Considering the hypotheses tested in the study, we will discuss the implications of the results and their alignment with the research objectives.

The results of our quantitative analysis provided empirical evidence to support the hypotheses H1 and H2 that there is a negative relationship between Afro-pop music and Yoruba youth cultural behaviour, which is also related to negative societal morality. The association between exposure to explicit content and the promotion of materialism, sexual promiscuity, and drug use among young individuals was found to be significant. This aligns with the observation of [35] that there is an association between time spent listening to music in general and changes in sexual behaviour. Not focusing solely on changes in sexual behaviour, [8] also stressed that sexism and vulgarity have become commonplace in the present-day Afro-pop musical works of notable artists such as Wizkid, Flavour, and Olamide, among others. Reuben [74] also asserts that Afro-pop music may shape attitudes, beliefs, and social interactions.

These findings underscore the importance of understanding the potentially harmful consequences of music content on impressionable minds and raises concerns about the need for appropriate regulation and content scrutiny. Therefore, it is crucial to take into account the wider societal consequences of music-influenced youth cultural behaviour. The findings also provided support for H3. The relationship between Afro-pop music lyrics and societal morality was found to be significant, and this show that 30.4% of the variance in morality in society variable is explained by afro pop music lyrics. This suggests that though Afro-pop music content may influence societal morality. Odetade and Fashanu [75] stress that the use of vulgarity in pop music raises concerns about its influence on moral values and societal norms. Some studies have suggested that exposure to this explicit content in music may contribute to the desensitisation of individuals, leading to behavioural changes and potential degradation of cultural values also present-day hip-hop music has deviated from fostering a sense of national awareness and addressing pressing matters concerning governmental policies [76].

However, there are other factors and influences that also play a role in shaping moral values within society. Future research could delve deeper into the complexities of this relationship to gain a more comprehensive understanding.

### 4.1 Acknowledging the multifaceted nature of Afro-pop music

Although the present research has concentrated on the critical issues of the so-called vulgar content in the Nigerian Afro-pop music, one must admit the fact that this genre is not entirely negative. Afro-pop plays a very significant positive cultural role, which should be considered. The genre has also helped popularise Nigerian culture worldwide, helped artists and the creative industry gain economic sustenance, enabled social commentary on political matters, and provided Nigerian youth in the diaspora with a sense of cultural pride and identity [12]. Wizkid, Burna Boy, and Davido are the ones who have achieved international success on unprecedented levels, introducing the African music culture to the world audience and proving the Western-controlled music industry discourse wrong, without marginalizing African culture, but making it even stronger [77].

Further, not all Afro pop music is made of clear and moral content. Most artists create their work in honor of the African culture, issues of social justice, positive messages, and traditional cultural themes without using vulgarity [78]. It is not that the genre needs to be demonized, but rather that it is necessary to promote critical media literacy among the young people who watch movies, and responsible content production among musicians without infringing artistic freedom and creativity. Youths who critically consume the contents of music, that is, they know that entertainment is an art that may not necessarily be true to life, are far less prone to internalising its negative effects than those who consume music in a naive manner, as observed by Oniwon and Salami [6] in their focus group.

The meaning of our findings must then be taken in the light of pointing out particular areas of concern in the context of a larger cultural phenomenon, which has its beneficial and even destructive aspects. The statistical data used in this paper prove that listening to vulgar lyrical materials is associated with such adverse cultural and ethical consequences among the Yoruba young people. The association, however, is not impossible or irreversible. It is not censorship and anti-creative action but rather an informed consumption, a culturally aware production, and a set of structural possibilities to allow for the positive aspects of Afro-pop to bloom as the young listeners are shielded from the more destructive effects of the movement.

## 5 Recommendations and conclusion

This study provides valuable insights into the cultural implications of vulgar Nigerian Afro-pop music on Yoruba youth and society. Considering results in this study and the discussed implications, various recommendation emerges to address the said negative relationships and encourage judicious content consumption among young individuals.

### 5.1 The role of regulatory bodies

As the genre enjoys popularity both within Nigeria and on the global stage, it becomes crucial for regulatory bodies to establish clear guidelines and standards to ensure that the content aligns with cultural values while safeguarding the well-being of young listeners. Striking a balance between artistic freedom and responsible content creation is of utmost importance.

### 5.2 The need for media literacy and education

Our study’s findings highlight the need for media literacy and education among Yoruba youth to navigate the influence of Afro-pop music effectively. By promoting critical thinking skills and responsible consumption of media, young individuals can be empowered to make informed decisions about their attitudes and behaviours, reducing the potential negative impact of explicit content.

### 5.3 Societal and policy implications

The research outcomes have significant societal and policy implications. By recognizing the potential negative impact of explicit content on Yoruba youth, policymakers, music industry stakeholders, and educators can work together to create a responsible and empowering music environment. This involves nurturing the positive aspects of Afro-pop music while addressing the concerns raised by critics to promote cultural pride and identity while safeguarding young listeners from harmful influences.

While the study provides valuable insights into the cultural implications of vulgar Afro-pop music, it is essential to acknowledge its limitations. The research focused on quantitative analysis, and future studies could complement these findings with qualitative approaches to gain a more nuanced understanding of the topic.

### 5.4 Limited support for the positive impact hypothesis

While our study primarily focused on exploring the negative impact of vulgar Afro-pop music, it is important to acknowledge that the findings provided limited support for the positive impact hypothesis. While Afro-pop music does promote Nigerian culture and identity, its positive influence on empowering Yoruba youth was not as strongly evident in our analysis. Future research may need to explore this aspect further to gain a more comprehensive understanding of the genre’s potential positive effects. In conclusion, the study provided valuable insights into the cultural implications of vulgar Nigerian Afro-pop music among Yoruba youth. The findings supported the hypothesis of negative impact, emphasizing the need for regulatory measures, media literacy, and responsible content creation. As Afro-pop music continues to be a powerful cultural force, it is crucial to harness its potential positively while being mindful of its influence on young minds and society as a whole.

## Supporting information

S1 Appendix Appendix A. (DOCX)
