# Supplementary material for: Exploring obscenity in Nigerian Afro-pop music: A sociocultural analysis of Yoruba youth and social norms
Source: PLoS One. 2026 May 5;21(5):e0346533. doi: 10.1371/journal.pone.0346533 (PMC13143061; doi:10.1371/journal.pone.0346533)
Supplement: S1 Appendix — (DOCX) [file pone.0346533.s001.docx]

**Appendix A: Constructs and Questionnaire**

| **Constructs** | **Items** | **Source (s)** |
| --- | --- | --- |
| Afro-Pop Lyrics | 1. I pay attention to the Afro-pop lyrics in Nigeria. 2. Afro-pop music from Nigeria frequently uses vulgar language. 3. I find it offensive when vulgar language is used in Nigerian Afro-pop music. 4. The Afro-pop lyrics from Nigeria play a significant role in influencing Yoruba youths' behavior. 5. The Afro-pop music from Nigeria accurately reflects the situation of the society in its lyrics. | [69] |
| Negative Influence | 1. Yoruba youths are negatively impacted by the vulgar language used in Nigerian Afro-pop music. 2. The use of vulgar words in Nigerian Afro-pop music has the potential to encourage Yoruba youths to violence. 3. The degrading of morals among Yoruba youths is a reflection of the vulgarity used in Nigerian Afro-pop music. 4. The vulgar language used in Nigerian Afro-pop music encourages a culture of disrespect among Yoruba youths. 5. The Afro-pop music from Nigeria contributes to the objectification of women by using vulgar language. | [70] |
| Morality in Society | 1. The vulgar language in Nigerian Afro-pop songs shows a lack of respect for Yoruba cultural values. 2. The vulgarity in Nigerian Afro-pop songs is a reflection of the larger cultural shift toward immorality. 3. The use of vulgar language in Nigerian Afro-pop is an indication that traditional Yoruba values are degrading. 4. To preserve Yoruba culture, the authorities must place regulations on the use of profanity in Nigerian Afro-pop music. 5. Nigerian Afro-pop artists have a societal obligation to spread ethical and moral behavior among Yoruba youth. | [71-72] |
